# A heterozygous mutation in UBE2H in a patient with developmental delay leads to an aberrant brain development in zebrafish

**DOI:** 10.1186/s40246-023-00491-7

**Published:** 2023-05-19

**Authors:** Unbeom Shin, Yeonsong Choi, Hwa Soo Ko, Kyungjae Myung, Semin Lee, Chong Kun Cheon, Yoonsung Lee

**Affiliations:** 1 School of Life Sciences, Ulsan National Institute of Science and Technology (UNIST), Ulsan, 44919 Republic of Korea; 2 Department of Biomedical Engineering, UNIST, Ulsan, 44919 Republic of Korea; 3 Korean Genomics Center, UNIST, Ulsan, 44919 Republic of Korea; 4 Center for Genomic Integrity, Institute for Basic Science (IBS), Ulsan, 44919 Republic of Korea; 5 Division of Medical Genetics and Metabolism Department of Paediatrics, Pusan National University School of Medicine, Pusan National University Children’s Hospital, Yangsan, 50612 Republic of Korea; 6 Research Institute for Convergence of Biomedical Science and Technology, Pusan National University Yangsan Hospital, Yangsan, 50612 Republic of Korea; 7 Clinical Research Institute, Kyung Hee University Hospital at Gangdong, College of Medicine, Kyung Hee University, Seoul, 05278 Republic of Korea

**Keywords:** UBE2H, Rare disease, Zebrafish, Transcriptomics, Brain, p53, ATM

## Abstract

**Background:**

Ubiquitin-related rare diseases are generally characterized by developmental delays and mental retardation, but the exact incidence or prevalence is not yet fully understood. The clinical application of next-generation sequencing for pediatric seizures and developmental delay of unknown causes has become common in studies aimed at identification of a causal gene in patients with ubiquitin-related rare diseases that cannot be diagnosed using conventional fluorescence in situ hybridization or chromosome microarray tests. Our study aimed to investigate the effects of ubiquitin–proteasome system on ultra-rare neurodevelopmental diseases, through functional identification of candidate genes and variants.

**Methods:**

In our present work, we carried out genome analysis of a patient with clinical phenotypes of developmental delay and intractable convulsion, to identify causal mutations. Further characterization of the candidate gene was performed using zebrafish, through gene knockdown approaches. Transcriptomic analysis using whole embryos of zebrafish knockdown morphants and additional functional studies identified downstream pathways of the candidate gene affecting neurogenesis.

**Results:**

Through trio-based whole-genome sequencing analysis, we identified a de novo missense variant of the ubiquitin system-related gene *UBE2H* (c.449C>T; p.Thr150Met) in the proband. Using zebrafish, we found that Ube2h is required for normal brain development. Differential gene expression analysis revealed activation of the ATM-p53 signaling pathway in the absence of Ube2h. Moreover, depletion of *ube2h* led to induction of apoptosis, specifically in the differentiated neural cells. Finally, we found that a missense mutation in zebrafish, *ube2h* (c.449C>T; p.Thr150Met), which mimics a variant identified in a patient with neurodevelopmental defects, causes aberrant Ube2h function in zebrafish embryos.

**Conclusion:**

A de novo heterozygous variant in the *UBE2H* c.449C>T (p.Thr150Met) has been identified in a pediatric patient with global developmental delay and UBE2H is essential for normal neurogenesis in the brain.

**Supplementary Information:**

The online version contains supplementary material available at 10.1186/s40246-023-00491-7.

## Background

The ubiquitin–proteasome system, which is associated with multiple central nervous system disorders, plays a critical role in the processing of damaged proteins or toxic substances involved in various neurodegenerative disorders [1]. The typical symptoms of ubiquitin-related rare diseases are growth disorder, cognitive developmental abnormalities, language delay, and convulsions in patient, most of which occur at a young age. Microcephaly and seizures are common symptoms seen in case of mutations in ubiquitination-related genes, with developmental delay first detected as early as between 3 and 6 months of age; unique clinical features, however, do not appear until after 1 year of age [2].

Several patients with Angelman syndrome have mutations in the 15q11-13 *UBE3A* gene [3]. *UBE3A* is regulated by imprinting, such as methylation, in the brain or spinal cord, with its spatial expression pattern differing in each part of the brain [4, 5]. Dysfunction of UBE3A induces accumulation of downstream targets Arc and Ephexin-5 in the primary hippocampal neurons of the rat brain, leading to the internalization of GluR1 and activation of RhoA [6]. As a result, the loss of UBE3A leads to dysfunction and loss of brain synapses, which can cause neurodegenerative diseases. In addition, TRIM ubiquitin E3 ligase-mediated ubiquitination regulates various cellular processes, and mutations in TRIM family genes have been reported to cause developmental defects, neurodegeneration, and cancer [7, 8]. Especially, mutations in TRIM32 and TRIM-like malin have been reported to be associated with limb-girdle muscular dystrophy type R8 and Lafora disease, respectively [9].

Vourc'h et al. reported *UBE2H,* a member of the ubiquitin–proteasome system, as a candidate gene for autistic disorders [3]. UBE2H is an E2 ubiquitin-conjugating enzyme that delivers ubiquitin from the E1 ubiquitin-activating enzyme to the E3 ubiquitin ligase, to ubiquitinate target proteins for degradation [10, 11]. The *UBE2H* gene is located in the imprinted MEST region of 7q32 [12]. Although most imprinted genes are clustered in ‘imprinted regions’ as observed in regions 15q11-q13 (the *UBE3A* locus) or 11p15.5, the function of *UBE2H* as an imprinted gene is still not known in humans. UBE2H acts on the ubiquitination of histones and cytoskeletal proteins related to motor neuron degeneration pathways, and multiple variants have been reported in patients with amyotrophic lateral sclerosis (ALS) [13]. In addition, an increase in the transcription level of *UBE2H* has been reported in the blood of patients with Alzheimer’s disease [14]. Although the involvement of *UBE2H* in neurological diseases has been reported previously, there is still a lack of understanding of the gene’s function at the cellular and organismal levels.

In the present study, we found a novel mutant variant of the *UBE2H* gene through genome analysis of patients with clinical phenotypes of developmental delay and intractable convulsions. To understand novel ubiquitin-related mutations and their effects, we investigated the function of Ube2h during embryonic neurogenesis in zebrafish. Knockdown of *ube2h* using morpholino (MO) led to an abnormal brain size, caused by activation of the ATM-p53 signaling pathway. Cell death events were specifically induced in differentiated neural cells of the brain, through activation of p53 signaling. Finally, we confirmed that the variant of *UBE2H* (c.449C>T, p.Thr150Met) from the patient was not normally functional, through rescue experiments using the variant of zebrafish *ube2h*. The discovery of novel *UBE2H* mutations in patients and elucidation of the function of *UBE2H* in neurodevelopment will provide a better understanding of the effects of the ubiquitin–proteasome system on rare neurological diseases.

## Methods

### Clinical data collection

Informed consent was obtained from the patients, for publication and genetic analyses. This study was performed in accordance with the Declaration of Helsinki. A blood sample was collected for DNA extraction, and informed consent was obtained from all study participants before the blood was drawn. Data on demographics and other clinical features were collected from the clinical records of the patients.

### Whole-genome sequencing (WGS) of patient samples

The genomic DNA was fragmented using Frag enzyme (MGI, Shenzhen, China) into fragments between 100 and ∼1000 bp in size, which are suitable for PE150 sequencing, according to the manufacturer's instructions (MGI FS DNA library prep set, cat No. 1,000,005,256). The fragmented DNA was selected to be between 300 and 500 bp using DNA clean beads (MGI). The selected DNA fragments were then repaired to obtain a blunt end and modified at the 3′-end to obtain dATP as a sticky end. The dTTP-tailed adapter sequence was ligated at both ends of the DNA fragments. The ligation product was then amplified for 7 cycles and subjected to the following single-strand circularization process. The PCR product was heat-denatured with a special molecule that was reverse-complemented to one special strand of the PCR product, and the single-strand molecule was ligated using DNA ligase. The remaining linear molecule was digested with an exonuclease to obtain a single-strand circular DNA library. We sequenced the DNA library using DNBSEQ-T7 (DNBSEQ-T7, RRID:SCR_017981) with a PE read length of 150 bp. We also used FastQC v0.11.8 (FastQC, RRID:SCR_014583) to assess the overall sequencing quality of the MGI sequencing platforms. Sequencing reads were aligned to the human reference genome (GRCh38) using bwa (version 0.7.15), with the “-M” option [15]. Point mutations (SNVs/indels) were identified using HaplotypeCaller with the “-ERC GVCF” option, and jointly genotyped using the GenotypeGVCFs tool of GATK (version 4.1.7.0) [16]. The initially identified variants were recalibrated using the VariantRecalibrator and ApplyRecalibration tools. The recalibrated variants were further annotated based on the American College of Medical Genetics and Genomics (ACMG) [17] category, with a custom pipeline, using InterVar (version 2.2.2) [18]. The annotated variants were classified according to four different inheritance models: (1) de novo, (2) autosomal recessive, (3) compound heterozygous, and (4) X-linked recessive mutation. The variants belonging to each category were filtered on the basis of allele frequency (< 0.01) from large-scale variome databases (1000 Genomes Project [19], gnomAD [20], and Korea1K [21]), to remove common variants. The variants were also annotated using the resources provided by the Human Phenotype Ontology (HPO) [22].

### Validation of Sanger sequencing

Sanger sequencing was used to confirm the candidate variants and define their inheritance mode via familial segregation testing. All candidate variants were sequenced bidirectionally using the ABI PRISM 3.1 BigDye™ Terminator Kit (Applied Biosystems, Foster City, CA, USA). The sequencing products were resolved on an ABI PRISM 3130XL sequencer (Applied Biosystems), and the chromatograms were analyzed using the Sequencer 4.9 software (Gene Codes, Ann Arbor, MI, USA). The mutation nomenclature was based on the cDNA reference sequence for the *UBE2H* (NM_001202498) gene.

### Protein structural modeling

Protein structural modeling analysis was performed for the case of UBE2H. The crystal structures of the domains of wild-type UBE2H were generated using SWISS-MODEL (https://swissmodel.expasy.org/). All structural images were generated using PyMOL, a molecular visualization software 29 [DeLano WL. The PyMOL Molecular Graphics System; 2002. San Carlos, CA, USA: DeLano Scientific. Available from: https://pymol.org/2/].

### Zebrafish husbandry

All embryo and adult zebrafish animals were raised in the circulating aquarium system (Genomic Design, Daejeon, Republic of Kore) at 28.5 °C, in accordance with Ulsan National Institute of Science and Technology and Use Committees (IACUC: UNISTIACUC-20-09). The wild-type (WT) TAB strain was used for MO or mRNA injection experiments, while *tp53*^zdf1/zdf1^ line was used for loss-of-function studies on p53. Tg(*ngn*:RFP) and Tg(*huc*:DsRed) lines were used for fluorescence imaging.

### Microinjection of MO and mRNA

One-cell stage embryos were injected with 1 nl of antisense *ube2h* MO (GeneTools, Philomath, OR, USA) and/or *ube2h* mRNA. *ube2h* MO-5’-ACTCTCGATGCTAAAGGAAGAATGT-3’ was used at a concentration of 2.5 ng/nl. *ube2h* mRNA was synthesized from linearized pCS2p+ vectors using the mMESSAGE mMACHINE™ SP6 Transcription Kit (Invitrogen, Waltham, MA, USA) and used at a concentration of 100 pg/nl. A FemtoJet 4i microinjector (Eppendorf, Hamburg, Germany) was used for microinjection with borosilicate glass needles (Sutter Instrument, Novato, CA, USA) fabricated on a PMP-102 Micropipette Puller (MicroData Instrument, Plainfield, NJ, USA).

### Whole-transcriptome sequencing of zebrafish morphants

We used 100 ng total RNA from all subjects to prepare sequencing libraries with by using the TruSeq-stranded total RNA sample preparation kit (Illumina, CA, USA) which combines RiboZero rRNA depletion with a stranded-specific method similar to the dUDP method. Quality of these cDNA libraries was evaluated with the Agilent 2100 BioAnalyzer (Agilent, CA, USA). They were quantified with the KAPA library quantification kit (Kapa Biosystems, MA, USA) according to the manufacturer’s library quantification protocol. Following cluster amplification of denatured templates, sequencing was progressed as paired-end (2 × 150 bp) using Illumina NovaSeq6000 platform. To calculate gene expression levels, we used the RSEM pipeline (version 1.3.0) [23] with the zebrafish reference genome and transcriptome (GRCz11). Differential gene expression analysis was performed using DESeq2 (version 1.26.0) [24], with the default settings. Differentially expressed genes were identified as those that with a *p* value < 0.01 and absolute log2(fold change) > 1. Gene set enrichment tests were conducted using Enrichr [25], and enriched terms with an adjusted *p* value < 0.01 were identified based on the Gene Ontology (GO) and Biological Process (BP) databases [26].

### *p53* mutant genotyping

Whole embryos or adult zebrafish fin-clips were lysed by means of incubation in a mixture of 50 μl of Extraction Solution and 14 μl of Tissue Preparation Solution (Sigma-Aldrich, St. Louis, MO, USA) at room temperature for 10 min, 95 °C for 5 min, and 25 °C for 5 min. The samples were neutralized with 50 μl of Neutralization Solution (Sigma-Aldrich). Aliquots from these lysates were subjected to PCR using GoTaq Master Mix (Promega, Madison, WI, USA) with the primer sets (tp53_zdf1_forward: 5′-ACATGAAATTGCCAGAGTATGTGTC-3′, tp53_zdf1_reverse: 5′-TCGGATAGCCTAGTGCGAGC-3′). The PCR conditions used included: 94 °C (12 min), 35 cycles of amplification (94 °C for 30 s, 57 °C for 30 s, and 72 °C for 30 s), and a final extension at 72 °C for 10 min, followed by an indefinite hold at 4 °C. The amplicons thus obtained were sequenced to detect the point mutation.

### Whole mount in situ hybridization (WISH)

Zebrafish embryos were fixed using 4% paraformaldehyde at 4 °C. The fixed samples were washed using PBS and dehydrated using methanol at − 20 °C. After dehydration, the samples were rehydrated using PBS with 0.1% Tween-20 and permeabilized using acetone at − 20 °C. The permeabilized samples were hybridized with digoxigenin (DIG)-labeled RNA probes in hybridization buffer [50% formamide, 5 × SSC, 500 μg/ml Torula yeast tRNA, 50 μg/ml heparin, 0.1% Tween-20 and 9 mM citric acid (pH 6.5)]at 65 °C. After hybridization, the samples were washed using 2 × and 0.2 × SSC solutions. The washed samples were incubated with alkaline phosphatase-conjugated antibodies against DIG (1:5000, Roche, Basel, Switzerland) at 4 °C. For signal development, the samples were incubated in alkaline phosphatase reaction buffer [100 mM Tris (pH 9.5), 50 mM MgCl_2_, 100 mM NaCl, 0.1% Tween-20 and 1 mM tetramisole hydrochloride] with NBT/BCIP substrate (Promega).

### 5-Ethynyl-2′deoxyuridine (EdU) assay

Zebrafish embryos were incubated in an EdU solution [10 mM EdU in E3 fish water with 15% DMSO] for 10 min. The embryos were fixed using 4% paraformaldehyde at 4 °C, and dehydrated using methanol at − 20 °C. After dehydration, the samples were rehydrated using PBS with 0.1% Tween-20 (PBT) and permeabilized using PBS with 1% Triton X-100. EdU signals were detected using the Click-iT™ EdU Alexa Fluor™ 488 Imaging Kit (Invitrogen).

### Acridine orange (AO) staining

Zebrafish embryos were incubated in a 20 μg/ml AO solution dissolved in E3 fish water for 10 min. After washing twice with E3 water, the samples were immediately imaged using an LSM880 confocal microscope (Carl Zeiss).

### TUNEL assay

The zebrafish embryos were fixed using 4% paraformaldehyde at 4 °C and dehydrated using methanol, at − 20 °C. The dehydrated samples were rehydrated using PBT and permeabilized using PBS with 0.5% Triton X-100 and 1% DMSO. After permeabilization, the samples were post-fixed using ethanol:acetic acid (2:1) at − 20 °C, and blocked in PBS containing 5% BSA at 4 °C. The samples were washed using PBT and blocked with biotin using a Streptavidin/Biotin Blocking Kit (Vector Laboratories, Newark, CA, USA). After blocking, the samples were incubated in equilibration buffer (PBS with 1 × TdT reaction buffer and 1 × CoCl_2_; Roche), which was then replaced with TdT reaction solution (equilibration buffer with 600 units of Terminal Transferase and Biotin-16 UTP; Roche). For TUNEL signal development, the samples were incubated in PBT with streptavidin, Alexa Fluor™ 647-conjugated antibody (1:5000, Thermo Fisher Scientific, Waltham, MA, USA), and mounted in VECTASHIELD® Antifade Mounting Medium with DAPI (Vector Laboratories).

### Treatment with ATM inhibitor (ATMi)

*ube2h* morphant and uninjected control clutches were incubated with 200 μM ATMi (KU60019; Sigma-Aldrich) or 2% DMSO dissolved in E3 fish water from 6 to 24 h post fertilization (hpf). The embryos were immediately fixed using 4% paraformaldehyde for TUNEL assay.

### Microscopy and image analysis

Bright field imaging of live embryos or WISH samples was performed using an M165C digital stereo microscope (Leica, Wetzlar, Germany). An LSM880 confocal microscope was used to image the fluorescence transgenic embryos and the samples stained with EdU, AO and TUNEL assays.

### Site-directed mutagenesis

To change ACA to ATG at the 448^th^–450^th^ nucleotide positions in the sequence of the zebrafish *ube2h* gene, we amplified a fragment of *ube2h* with two oligos (forward: 5′-ATGAACATCGATGAAGCGTCAGGGACCGTGTGTTTAGATG-3′, reverse: 5′- GGCTCGAGCTACAACTCCATGTCCTGAGCCTCGTCCTCTGAAAAGTCAGACATGGAGCTTTCTGAAGAGGAGTCGCCCGGTCCTTCCTCCTGCTCCTTCAGAGCCTCCTCCATTGCATATTTC-3′). The amplicons were ligated using restriction enzyme sites and transformed into the pCS2p+ vector.

## Results

### Clinical presentation

**Fig. 1:**
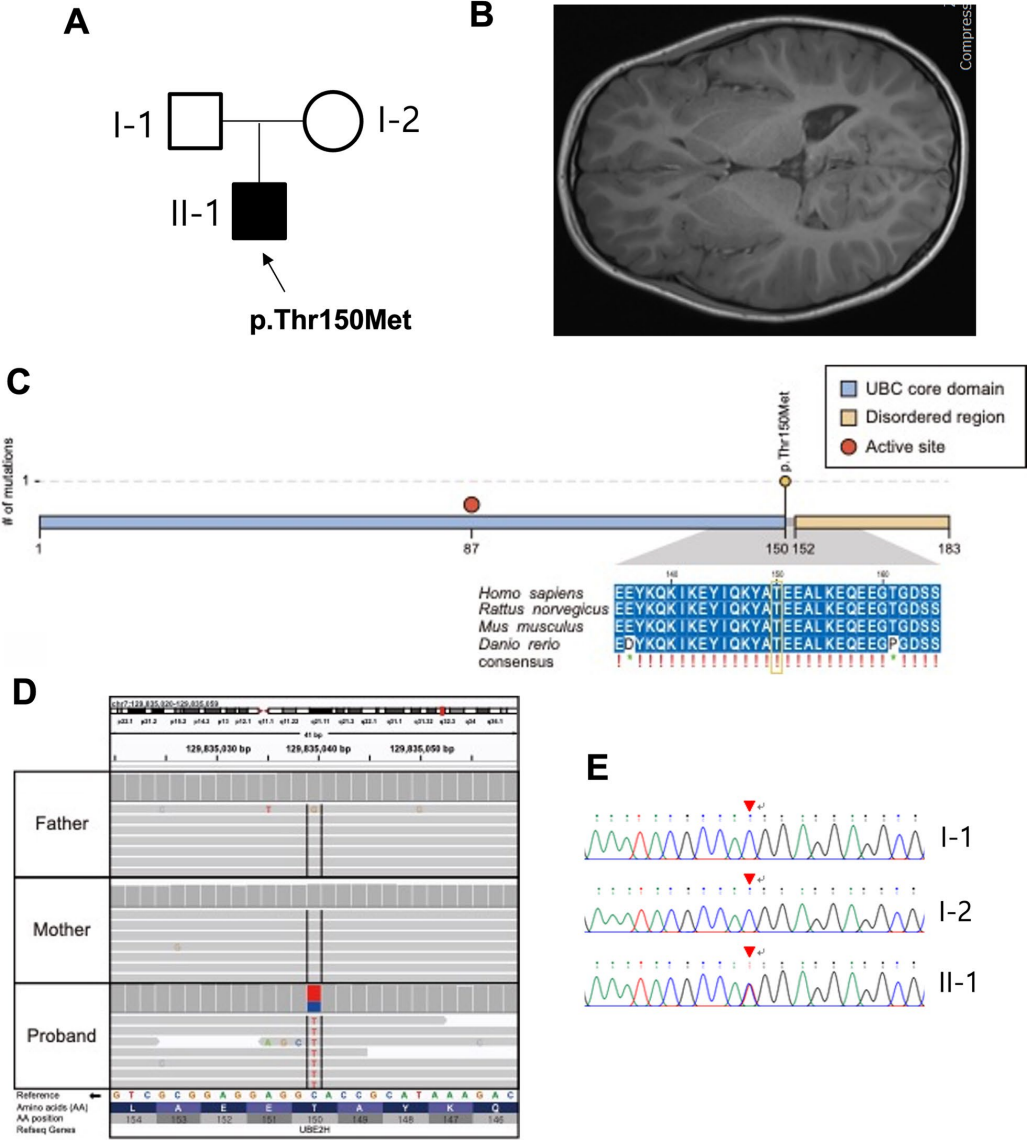
A de novo mutation of UBE2h was found in a patient with neural development defects in the brain. **A** Pedigree of the present family. The proband has been indicated using an arrow. The affected male has been designated using the filled symbol (squares for males and circles for females). **B** Brain magnetic resonance imaging (MRI) scans of the patient with dolichocephaly. **C** The variant (c.449C>T, p.Thr150Met) was located in the UBC core domain of UBE2H and was perfectly conserved in four model species according to multiple sequence alignment analyses. **D.** IGV snapshot of the variant (c.449C>T, p.Thr150Met). **E.** Sanger sequencing confirmed a heterozygous missense variant (c.887A>G, p.Thr150Met) of the *UBE2H* gene (NM_001202498) that had been identified using whole-genome sequencing of the affected patient genome (II-1)

The patient in our study was a 5-year-old male with an overall developmental delay. The patient was born to healthy and non-consanguineous parents with no family history (Fig. 1A). The patient had no specific perinatal history, although he was a preterm infant born at 36 + 4 weeks of gestation, with a low birth weight of 2.1 kg. He could barely walk until he was 2 years old. In the Bayley Scales of Infant and Toddler development conducted at the age of 2 years, the patient showed 18 months of recognition, 13 months of acceptance language, 15 months of expression language, 17 months of fine movement development, and 14 months of gross movement development. At about the age of three, he developed convulsions. He had generalized tonic–clonic seizures over several minutes. His seizures showed tonic–clonic movements with regular tremors of the limbs after a period of rigidity. Upon admission at the age of 5 years, the patient had a head circumference of 50 cm (− 1.10 standard deviation score (SDS)), a height of 104.5 cm (− 0.94 SDS), and a weight of 15.6 kg (− 1.55 SDS). The patient displayed relative macrocephaly, dolicocephaly (Fig. 1B), large ears, epicanthal fold, smooth philtrum, and an enlarged penile length. The patient also showed central hypotonia and calf tightness. Although Fragile X syndrome was initially suspected by the patient’s physical features, CGG expansion in *FMR1* was not observed in the patient.

The patient’s karyotype was normal (46, XY), and the array comparative genomic hybridization also showed no abnormalities. Interestingly, trio-based WGS performed using the genomic data of the patient and his parents revealed a de novo heterozygous novel variant, c.449C>T (p.Thr150Met), in the exon 7 of the *UBE2H* gene, which was characterized by the base substitution of C to T at nucleotide position 449. The p.Thr150Met mutation is located in the ubiquitin-conjugating (UBC) core domain of the UBE2H. Furthermore, the amino acid sequences of the variant locus were completely conserved across multiple species (Fig. 1C, D), and this variant was confirmed using Sanger sequencing (Fig. 1E). The p.Thr150Met mutation was identified as a rare variant of *UBE2H* based on the 1000 Genomes Project, gnomAD, and Korea1K databases (Additional file 1: Table S1).

**Fig. 2:**
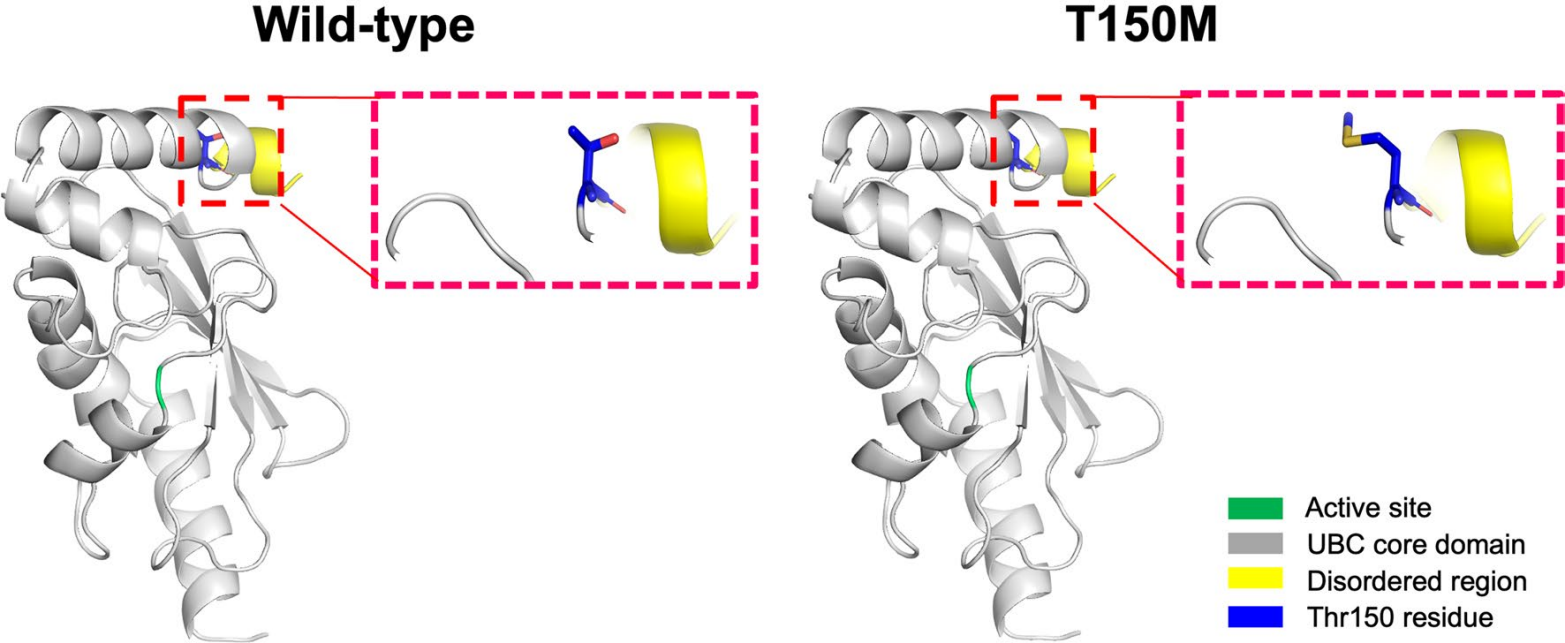
Structure homology-modeling of the normal human UBE2H and T150M variant. By means of in silico protein structure modeling, wild-type and mutant residues (p.Thr150Met) in the UBE2H protein have been represented as sticks alongside the surrounding residues. The Thr150 residue is located in the UBC core domain away from the active site. The crystal structure of the domain from wild-type UBE2H was generated using SWISS-MODEL (https://swissmodel.expasy.org/) and has been depicted as a cartoon representation

Simulation of protein crystallization modeling showed that the p.Thr150Met variant may affect the formation of weak hydrogen bonds between the surrounding residues, thereby possibly withering the activity of UBE2H (Fig. 2). Additional in silico analyses predicted the p.Thr150Met variant to be deleterious [SIFT (http:// sift.jcvi.org/) score = 0.04, prediction: damaging; PolyPhen (http://genetics.bwh.harvard.edu/pph/) score = 0.958, prediction: probable damaging; and MutationTaster (https://www.mutationtaster.org/) score = 0.999, prediction: disease-causing].

### Knockdown of *ube2h* causes defects in zebrafish brain neurogenesis

**Fig. 3:**
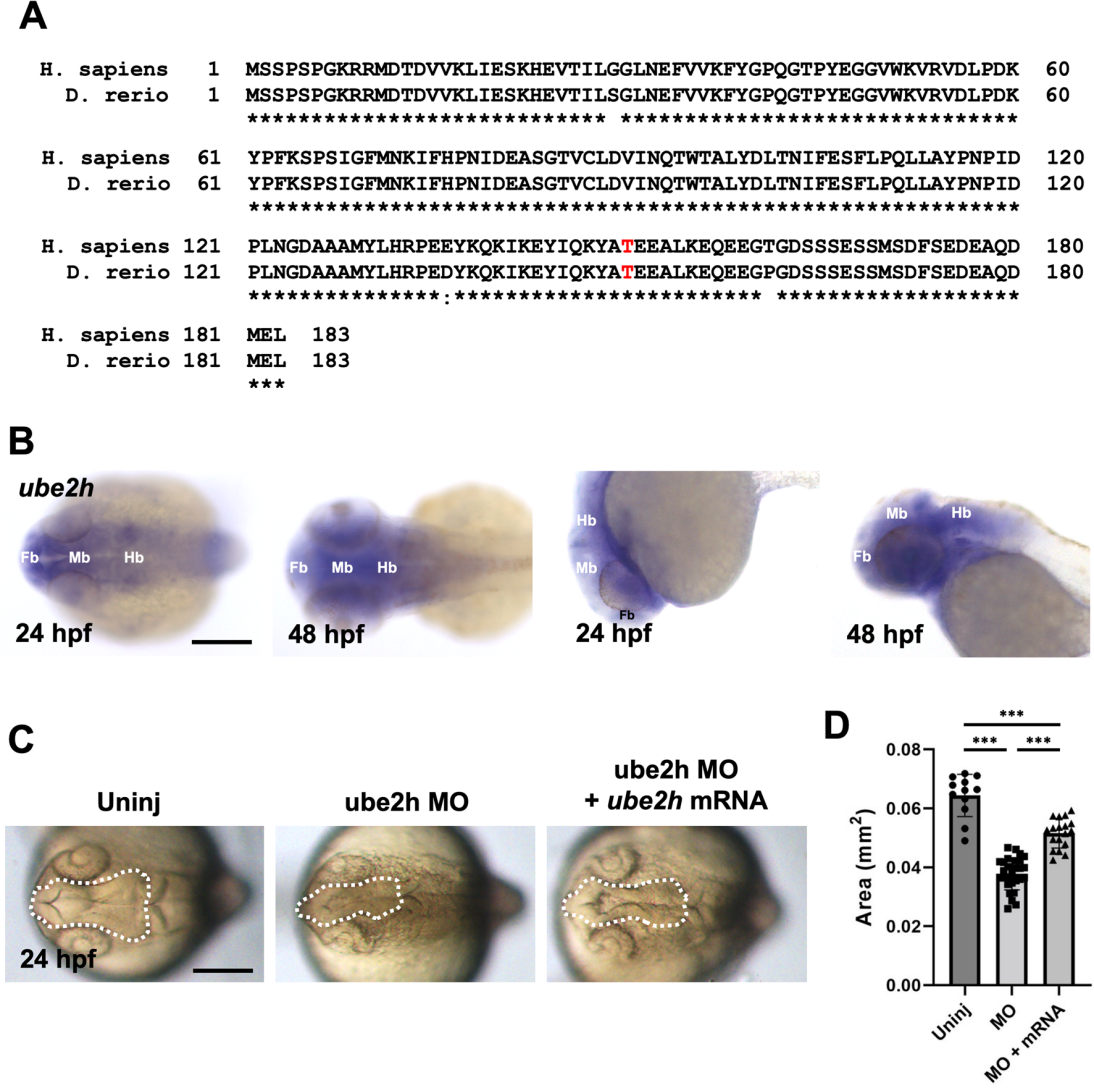
Ube2h is required for normal brain development. **A** Comparison of the amino sequences between human UBE2H and zebrafish Ube2h. The conserved sequences have been marked using an asterisk (*), while semi-conserved sequences have been marked using a colon (:). Thr150, where a missense mutation was found in the patient (Thr150Met), has been indicated in red. **B** Representative dorsal and lateral view images of *ube2h* WISH in the anterior region, at 24 or 48 hpf. *ube2h* is expressed in the anterior region, broadly including forebrain (fb), midbrain (mb), and hindbrain (hb). **C** Dorsal view of anterior region of *ube2h* morphant, *ube2h* morphant with *ube2h* mRNA injection and uninjected controls. The white dotted area indicates the brain tissue including forebrain and midbrain. **D** Quantification of the brain size from the dotted area in **C**. The graph represents mean ± S.E.M. of individual values. *p* values were calculated using an unpaired two-tailed Student’s *t* test. ***p < 0.001; *p < 0.05; and n.s., not significantly different. Scale bar: 200 μm

To further characterize the function of UBE2H in vivo*,* including in neural development, we used the teleost zebrafish as an animal model system. Zebrafish contain one ortholog of human *UBE2H* with a highly conserved amino acid sequence, especially Thr150 where the patient showed a de novo mutation (Fig. 3A). First, to examine the spatial distribution of *ube2h* during zebrafish embryogenesis, we performed WISH using an antisense probe of *ube2h*. *ube2h* was expressed in the anterior part of the zebrafish embryos including brain area including the forebrain, midbrain, and hindbrain areas at 24 and 48 hpf (Fig. 3B). Next, we performed knockdown analysis by injecting a splicing-block MO against *ube2h* (2.5 ng). The *ube2h* morphants showed an abnormal brain structure at 24 hpf, with a reduced size compared to that in the uninjected controls (Fig. 3C, D). Simultaneous injection of *ube2h* MO and normal *ube2h* mRNA restored the phenotypes of the *ube2h* morphant, which excludes the possibility of off-target effects of the *ube2h* MO on the brain-defective phenotypes of the morphants. Taken together, these data suggest that Ube2h is essential for normal brain development in zebrafish, which is consistent with the clinical phenotypes of the patient.

### p53-dependent apoptosis is induced in the brain in the absence of normal Ube2h function

**Fig. 4:**
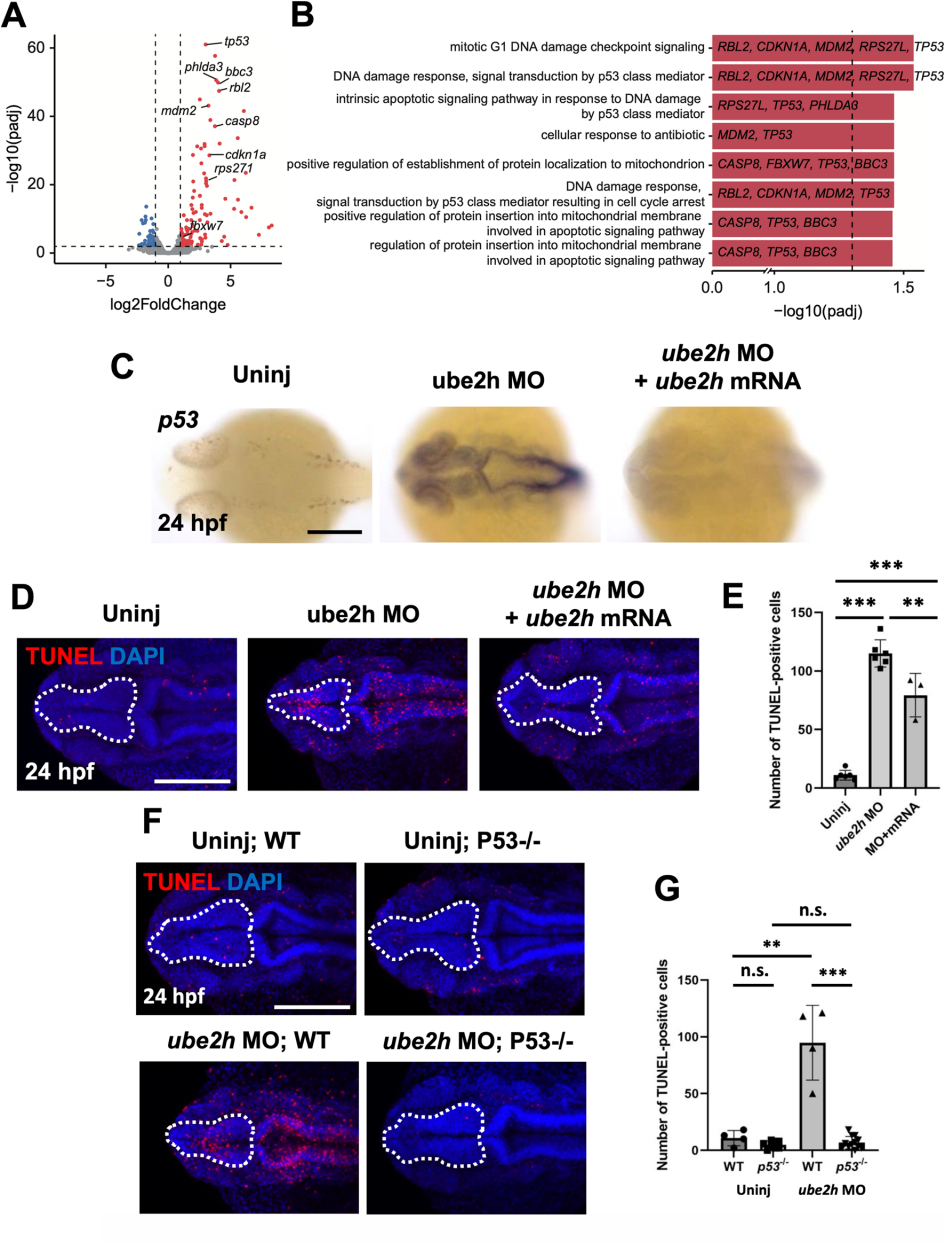
The p53-dependent apoptosis signaling pathway is highly activated in the *ube2h* morphant. **A** Volcano plot showing differentially expressed genes in the *ube2h* morphants. The red dots represent significantly upregulated genes, while blue dots represent downregulated genes in the *ube2h* morphants. **B** Visualization of the GO BP pathways significantly enriched with the differentially expressed genes in the *ube2h* morphants. **C** Dorsal view of *p53* WISH using *ube2h* MO-injected embryos, both *ube2h* MO and *ube2h* mRNA co-injected embryos, and uninjected controls, at 24 hpf. **D** Representative images of confocal microscopy of TUNEL staining in the brains of *ube2h* morphants, both *ube2h* MO and *ube2h* mRNA co-injected embryos, and uninjected controls at 24 hpf. **E** Quantification of the number of TUNEL-positive cells in the brains from **D**. **F** Confocal microscopy images of TUNEL-stained brains (white dotted area) of *ube2h* morphants and uninjected controls with either WT or *p53*^−/−^ background, at 24 hpf. **G** Quantification of the number of TUNEL-positive cells in the brains (white dotted area) from **F**. All graphs represent mean ± S.E.M. of individual values. *p* values were calculated using an unpaired two-tailed Student’s *t* test. *** *p* < 0.001; *p < 0.05; n.s., not significantly different. Scale bar: 200 μm

To identify biological processes associated with the disruption of brain development by the *ube2h* morphants, we compared RNA sequencing (RNA-seq) data from whole embryos of control and *ube2h* morphant zebrafish. We identified 122 significantly up-regulated genes and 59 down-regulated genes in the *ube2h* morphants, as compared to those in the controls (Fig. 4A). Gene Set Enrichment Analysis of those genes revealed that DNA damage response-related pathways such as ‘mitotic G1 DNA damage checkpoint signaling’ and ‘DNA damage response, signal transduction by p53 class mediator’ were significantly activated in the upregulated genes (Fig. 4B and Additional file 1: Fig. S1).

As expected from our transcriptomics analysis, WISH analyses showed that *p53* expression was highly induced in the brains of the *ube2h* morphants, whereas co-injection of *ube2h* mRNA with *ube2h* MO restored the level of *p53* expression as the uninjected controls (Fig. 4C). In addition, to examine cell death events possibly stimulated by the induced expression of *p53,* we performed apoptosis analysis using AO and TUNEL staining [27]. Consistent with the RNA-seq analysis, there was a profound increase in both AO- and TUNEL-positive cells in *ube2h* morphants (Fig. 4D, E; Additional file 1: Fig. S1B). We also confirmed that the highly induced apoptosis in the *ube2h* morphant was restored upon *ube2h* mRNA co-injection. Finally, to confirm whether apoptosis in the *ube2h* morphants was induced upon ectopic overexpression of *p53*, we knocked down *ube2h* in the *p53*^−/−^ mutant background. As expected, depletion of p53 restored the defective phenotypes in the *ube2h* morphants, including reduction in cell death in the brain and recovery of the brain size of the *ube2h* morphants (Fig. 4F, G; Additional file 1: Fig. S2). Taken together, these results suggested that p53-dependent cell death leads to abnormal brain development in the absence of normal Ube2h function.

Although antisense experimental approaches, including MOs, are highly efficient tools for functional studies, it is critical to validate their effects on phenotypes, to eliminate possible off-target effects [28]. One well-known off-target effects of MO injection is the induction of p53-dependent cell death [29]. To determine whether the phenotypes of the *ube2h* morphant were caused by direct knock-down of the *ube2h* gene or off-target effects, we performed rescue experiments using WT *ube2h* mRNA injection. In the morphants, WT *ube2h* mRNA injection restored *p53* expression and the number of apoptotic cells to the levels observed in the uninjected controls (Fig. 4C, D). These results suggested that *ube2h* MO knockdown successfully suppressed Ube2h, leading to the brain-defective phenotypes and ruling out possible p53-activated cell death as an off-target effect.

### Apoptosis of* ube2h* morphants is triggered by ATM-p53 signaling

**Fig. 5:**
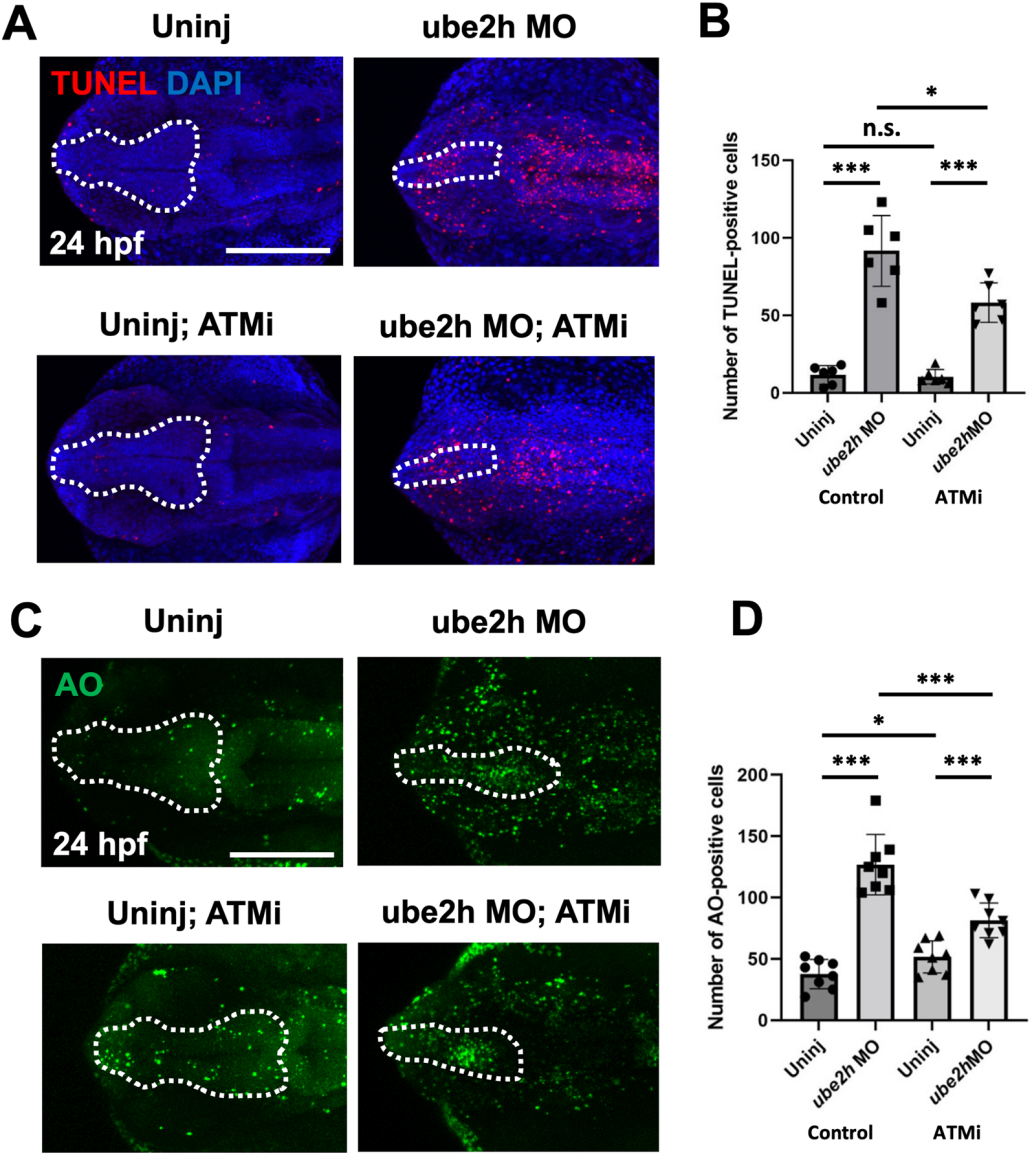
The ATM-p53 pathway activated apoptosis in the brain of *ube2h* morphants. **A** Representative confocal microscopy images of TUNEL-stained 24 hpf ube2h morphants treated with ATMi, KU60019. **B** Quantification of the number of TUNEL-positive cells in the brains (White dotted area) from **A**. **C** Confocal microscopy images of AO staining in the brains of 24 hpf *ube2h* morphants treated with ATMi. **D** Quantification of the number of AO-positive cells in the brains from **C**. The white dotted area indicates the brain area including forebrain and midbrain. The graphs represent mean ± S.E.M. of individual values. The *p* values were calculated using an unpaired two-tailed Student’s *t* test. ***p < 0.001; *p < 0.05; n.s., not significantly different. Scale bar: 200 μm. ATMi, ATM inhibitor

ATM signaling is a key DNA damage response that triggers apoptosis by activating the p53 pathways [30]. To determine whether ATM signaling is involved in p53-dependent apoptosis induced in *ube2h* morphants, we blocked ATM signaling in the *ube2h* morphants using an ATMi, KU60019. Consistent with our hypothesis, upon ATM inhibition, both TUNEL and AO staining showed a partial reduction in the number of apoptotic cells in the *ube2h* morphants (Fig. 5), suggesting that depletion of *ube2h* leads to the activation of ATM-p53 signaling, eventually inducing apoptosis in the brain.

### Ube2h is required for the survival and maintenance of neurons during embryogenesis.

**Fig. 6:**
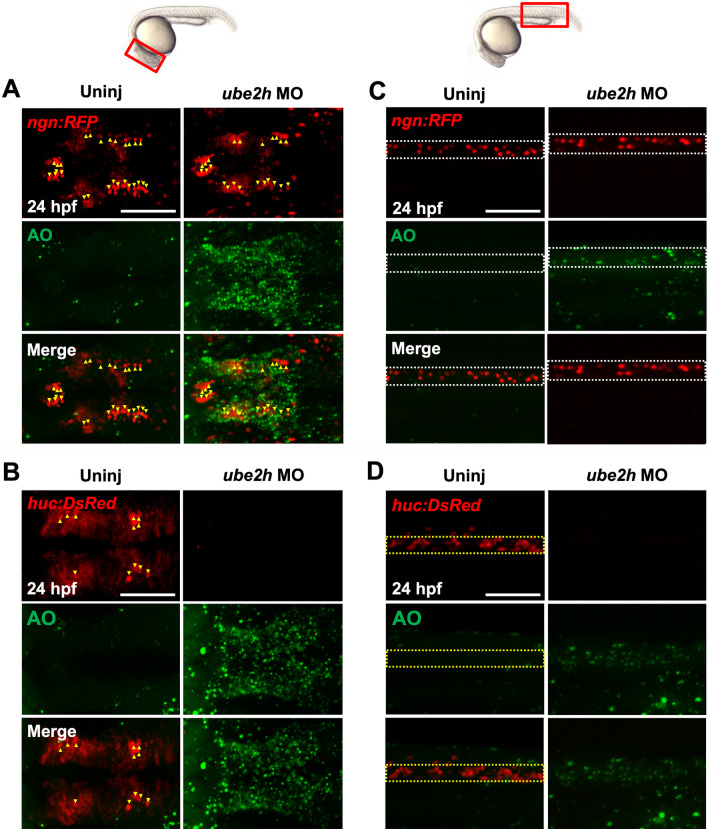
Depletion of *ube2h* disrupts the maintenance of neurons during zebrafish embryogenesis. **A**, **B** Dorsal view of confocal microscopy images of AO staining (Green) of the brains of *ube2h* morphants and uninjected controls with the transgenic background of either *ngn*:*RFP* (**A**) or *huc:DsRed* (**B**). at 24 hpf. Yellow arrowheads indicate either *ngn*- or *huc*-positive cells (red). **C**, **D** Lateral view of confocal microscopy images of AO staining in the body trunk of *ube2h* morphants and uninjected controls with the *ngn*:*RFP* (**C**) and *huc:DsRed* (**D**) transgenic background. The white dotted box indicates the *ngn*-positive dorsal area, while yellow bracket indicates the *huc*-positive ventral area in the neural tube

To further understand the effects of ATM-p53-dependent apoptosis in the absence of Ube2h activity, we labeled AO-positive apoptotic cells in the transgenic reporter lines with distinct neural cell types. *ngn:RFP* and *huc:DsRed* mark undifferentiated precursors with more initial stages of neurogenic cells and fully differentiated neurons, respectively, in zebrafish [31, 32]. Interestingly, *ngn*+ precursor cells formed normally in the *ube2h* morphants and AO+ apoptotic cells did not colocalize with *ngn*+ cells, whereas *huc*+ differentiated neurons failed to form in the brains of the *ube2h* morphants (Fig. 6A, B). Similar to that in the brain, *huc*+ neurons were abolished in the neural tissue of the body trunk without *ube2h*, while the formation of *ngn*+ precursors was not altered in the *ube2h* morphants. Furthermore, we performed WISH using *ube2h* morphants with distinct neural markers: *sox2* for neural stem cells, *neurod4* for intermediate neural progenitors in the middle stage of differentiation, and *huc* for fully differentiated neurons [33, 34]. Consistent with the results in the transgenic lines, there was a decrease in the expression of *neurod4* and *huc* in the brains of the morphants, whereas *sox2* expression was not altered (Additional file 1: Fig. S3). Taken together, our data suggest that Ube2h is required for the survival and maintenance of fully differentiated neurons during zebrafish embryogenesis.

### The zebrafish Ube2h variant Thr150Met showed loss-of-function

**Fig. 7:**
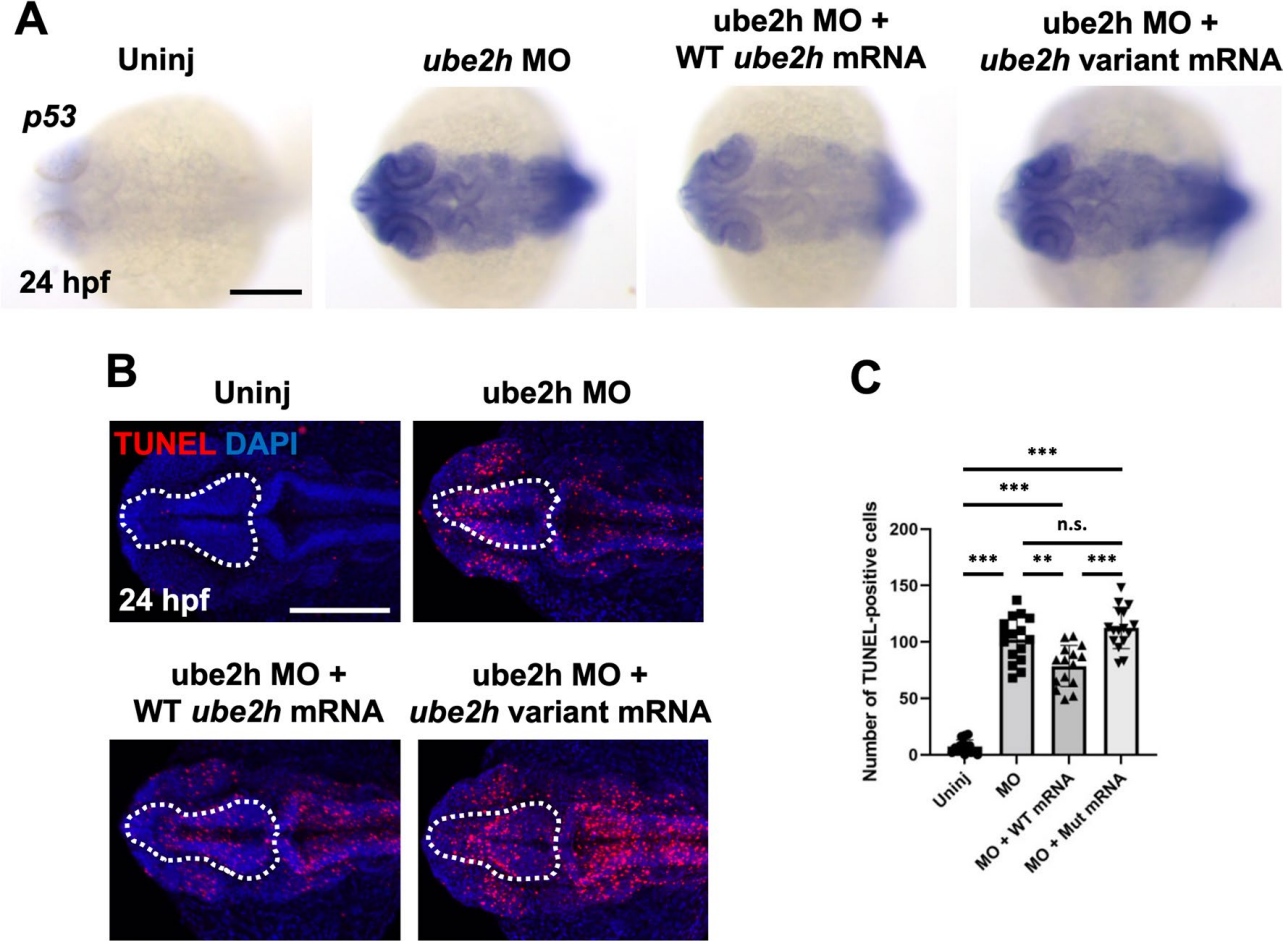
A Threonine to Methionine mutation at the 150th amino acid of Ube2h disrupts regulation of p53-dependent apoptosis. **A** Induction of *p53* expression in the *ube2h* morphant was recovered upon co-injection of WT *ube2h* mRNA with MO, but not upon co-injection of patient-mimicking *ube2h* variant mRNA with MO. **B** Representative confocal microscopy images of TUNEL staining in the brains of 24 hpf WT embryos injected with *ube2h* MO, *ube2h* MO with WT *ube2h* mRNA, and *ube2h* MO with patient-mimicking *ube2h* variant mRNA. **C** Quantification of the number of TUNEL-positive cells in the brains from **B**. All graphs represent mean ± S.E.M. of individual values. *p* values were calculated using an unpaired two-tailed Student’s *t* test. ***p < 0.001; **p < 0.01; n.s., not significantly different. Scale bar: 200 μm

Based on in silico analyses, the human missense *UBE2H* p.Thr150Met variant has been predicted to be deleterious. To examine whether the UBE2H variant is functionally altered, we generated the mRNA of the zebrafish *ube2h* variant Thr150Met and investigated its function in neurogenesis. Overexpression of the *ube2h* variant as well as WT *ube2h* alone did not alter brain developmental (Additional file 1: Fig. S4), indicating that the *ube2h* variant has no dominant-negative effects. To further validate the function of the variant, we performed rescue experiments with simultaneous injection of *ube2h* MO and the mutant variant mRNA. Interestingly, ectopically induced *p53* expression was not restored upon co-injection of *ube2h* MO and variant mRNA, whereas normal WT mRNA reduced *p53* transcription in the *ube2h* morphants (Fig. 7A). Likewise, the increase in the number of apoptotic cells in the absence of Ube2h was not recovered upon overexpression of the *ube2h* variant (Fig. 7B). Taken together, these results demonstrate that the variant Thr150Met found in human patient is hypomorphic or null, and can be deleterious, since it displayed abnormal function in both the patient and the experimental model.

## Discussion

Ube2h belongs to the E2 enzyme family, which is structurally and functionally conserved within multiple species [35]. In humans, UBE2H is expressed in neurons, blood, and muscles, and its activity is regulated by cytokine signaling, such as tumor necrosis factor-alpha (TNFa)/nuclear factor kappa b (NFkB) in skeletal muscles [14, 36]. The ubiquitin proteasome pathway has been shown to be associated with motor neuron death in ALS, the most frequent motor neuron disease in adults [13]. Furthermore, recent genetic studies have shown that Ube2h is associated with both brain development and human brain disorders, such as autism [3, 37], suggesting that *UBE2H* is highly polymorphic and mutations within *UBE2H* can affect neurodegenerative disorders.

This is the first study to report an ubiquitin pathway gene de novo heterozygous variant in the *UBE2H* gene, c.449C>T (p.Thr150Met), in a pediatric patient with global developmental delay, according to Online Mendelian Inheritance in Man (OMIM). Functional approaches carried out using zebrafish demonstrated that Ube2H is required for normal brain formation, and that the Ube2H variant p.Thr150Met is a loss-of-function mutation. Further transcriptomic analysis revealed that the induction of p53-dependent apoptosis leads to brain malformation in the absence of normal Ube2h. Compared to the macrocephaly phenotypes of the patient, knockdown of *ube2h* led to regressed brain with induced apoptosis in zebrafish. Variations in brain developmental phenotypes among hypomorphic and null alleles have been observed in other examples. While *CHD8* haploinsufficiency caused macrocephaly, homozygous deletion of *Chd8* leads to p53 activation and regression in the mouse brain, in a dosage-sensitive manner [38]. Likewise, knockdown of *ube2h* likely induced brain malformation via p53-dependent apoptosis in zebrafish, unlike the macrocephalic phenotypes observed in the patient with a heterozygous UBE2H variant.

Due to the high proliferative capacity of neural cells during development, the regulation of genome stability maintenance and DNA damage response is crucial for normal neurogenesis. Therefore, defects in genome integrity often result in neurological abnormalities [39–41]. E2 ubiquitin-conjugating enzymes, such as UBE2I, UBE2K, and UBE2N, play important roles in maintaining genome stability through various mechanisms including mitosis, chromosome stability, telomere maintenance, transcriptional regulation, and DNA damage repair [42]. Activation of ATM signaling leading to p53 stabilization is one well-known example in response to DNA damage. Similar to the roles of other E2 enzymes in maintaining genome stability, we found that apoptosis was highly induced by ATM-p53 activation in the *ube2h* morphants. The recovery of cell apoptosis through ATM inhibition, along with the activation of the p53 signaling pathway under *ube2h* knockdown conditions, suggest that Ube2h may play a critical role as one the E2 ligases in genome maintenance.

Proper regulation of p53 is essential for neural differentiation and neuronal survival [43, 44]. ATM also phosphorylates MDM2, MDMX and CHK2 leading to further stabilization of p53 through their interaction [45]. The activity of p53 signaling is regulated by several E3 ubiquitin ligases, with MDM2 being the predominant enzyme involved in this process [46, 47]. Various E2 enzymes, such as UBE2D1, UBE2D2, UBE2D3 and UBE2K, have been shown to participate in MDM2-mediated ubiquitination [48]. Topors is another E3 ligase that can ubiquitinate p53 with specific E2 enzymes [49]. Moreover, ubiquitination of MDMX, ATM and Chk2 was also found through interaction with MDM2, COP1 and CUL1-containing E3 ligase complex respectively [50–52]. However, the associated roles of UBE2H with these E3 ligases is still not known. Since our results using zebrafish demonstrated a reduction in the mRNA level of p53 in *ube2h* morphants, it is possible that UBE2H may affect proteins involved in regulating p53 transcription, such as HuR, WIG1 and PARN [53]. Further molecular investigations are needed to identify the target E3 enzymes of UBE2H and understand their mechanism regulating the p53 pathway.

The ubiquitin–proteasome system plays important roles in neurogenesis, including the regulation of neural precursor proliferation, neural differentiation and maturation [54]. Distinct E3 ubiquitin ligases are specifically important at each stage of neurogenesis. While SCF^βTRCP^ controls the neuronal cell fate of stem cells, Huwe1 regulates the cell cycle exit of neural precursor cells, maintaining their proliferation [55, 56]. To promote neurogenesis, TRIM32 targets c-Myc and TRIM11 regulates the expression of Pax6 levels [57, 58]. Cdh1-APC controls the growth and patterning of axons, while Cdc20-APC regulates dendrite morphogenesis in neurons [59, 60]. Interestingly, our results showed that most apoptotic cells in *ube2h* morphants were differentiated neurons, and neural progenitor cells were unaffected. Moreover, the expression of post-mitotic neuron markers decreased without *ube2h,* while that of neural stem cell markers remained unchanged. Although the distinct roles of E3 ligases at each stage of neurogenesis are well characterized, their regulation by E2 conjugating enzymes remains unclear. Our functional studies suggest that UBE2H is more involved in neuronal maintenance and maturation than in progenitor regulation.

## Conclusions

Our data suggest that Ube2h is essential for normal brain development, especially for neuronal differentiation and survival. Loss of Ube2h leads to activation of the ATM-p53 signaling to specifically induce apoptosis in the differentiated neural cells. A de novo missense variant *UBE2H* (c.449C>T; p.Thr150Met) identified in a patient with neurodevelopmental defects is loss-of-function and causes aberrant Ube2h function on zebrafish neurogenesis. Further molecular and biochemical studies are needed to understand molecular mechanisms of UBE2H targeting certain E3 ligases to regulate neuronal differentiation.

## Supplementary Information

**Additional file 1.** Supplemental Figures and Tables.

## Data Availability

The whole-genome and whole-transcriptome sequencing data were deposited to SRA under the BioProject accession no: PRJNA915203.

## Abbreviations

UBE2H: Ubiquitin conjugating enzyme E2 H
ATM: Ataxia telangiectasia mutated
UBE3A: Ubiquitin-protein ligase E3A
GluR1: Glutamate receptor 1
RhoA: Ras homolog family member A
TRIM: Tripartite motif containing
MEST: Mesoderm-specific transcript
ALS: Amyotrophic lateral sclerosis
MO: Morpholino
PCR: Polymerase chain reaction
ACMG: American College of Medical Genetics and Genomics
HPO: Human phenotype ontology
WT: Wild-type
RSEM: RNA-seq by expectation–maximization
GO: Gene ontology
BP: Biological process
PBS: Phosphate-buffered saline
DIG: Digoxigenin
SSC: Saline-sodium citrate
NBT: Nitro blue tetrazolium
BCIP: 5-Bromo-4-chloro-3-indolyl-phosphate
EdU: 5-Ethynyl-2′deoxyuridine
AO: Acridine orange
TUNEL: Terminal deoxynucleotidyl transferase dUTP nick end labeling
DMSO: Dimethyl sulfoxide
ATMi: ATM inhibitor
DAPI: 4′,6-Diamidino-2-phenylindole
SDS: Standard deviation score (SDS)
WISH: Whole-mount in situ hybridization
FMR1: Fragile X messenger ribonucleoprotein 1
UBC: Ubiquitin-conjugating
RNA-seq: RNA sequencing
Sox2: Sex determining region Y-box 2
Neurod4: Neuronal differentiation 4
TNFa: Tumor necrosis factor-alpha
NFkB: Nuclear factor kappa b
OMIM: Online Mendelian Inheritance in Man
*CHD8*: Chromodomain helicase DNA binding protein 8
*Mdm2*: Mouse double minute 2
SCF^βTRCP^: A multi-subunit E3 ubiquitin ligase consisting of Cul1, Rbx1, Skp1, and the F-box protein βTrCP
Huwe1: HECT, UBA and WWE domain containing E3 ubiquitin protein ligase 1
*Cdh1-APC*: Cdc20 homolog 1-anaphase-promoting complex
*Cdc20-APC*: Cell division cycle protein 20-anaphase-promoting complex
MRI: Magnetic resonance imaging
Fb: Forebrain
Mb: Midbrain
Hb: Hindbrain
