# Supplementary material for: A heterozygous mutation in UBE2H in a patient with developmental delay leads to an aberrant brain development in zebrafish
Source: Hum Genomics. 2023 May 19;17:44. doi: 10.1186/s40246-023-00491-7 (PMC10199504; doi:10.1186/s40246-023-00491-7)
Supplement: Supplementary file 1 — Additional file 1. Supplemental Figures and Tables. [file 40246_2023_491_MOESM1_ESM.pdf]

SUPPLEMENTARY INFORMATION

Supplemental Figures

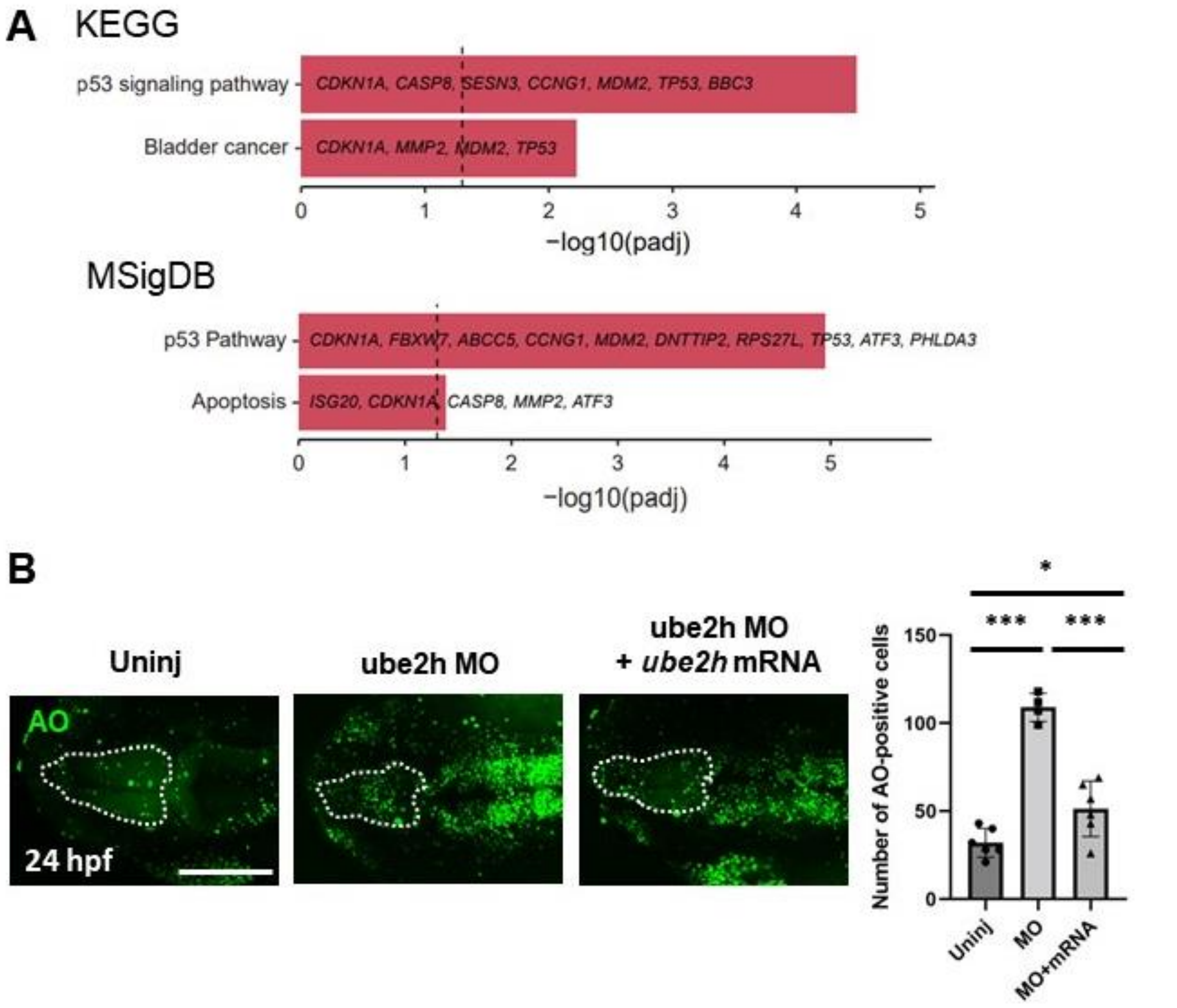

Supplemental Figure S1. RNA-seq of *ube2h* morphants revealed the induction of p53-dependent apoptosis in the absence of *ube2h*. A. GO analysis of RNA-seq results of *ube2h* morphants, as compared to those of uninjected controls. **B.** Representative images of confocal microscopy of AO staining in the brains of *ube2h* morphants, both *ube2h* MO and *ube2h* mRNA co-injected embryos, and uninjected controls, at 24 hpf and quantification of the number of AO-positive cells in the brain. All graphs represent mean ± S.E.M. of individual values. *p*-values were calculated using an unpaired two-tailed Student's t-test. *** $p<0.001$; *$p<0.05$; n.s., not significantly different. Scale bar: 200μm.

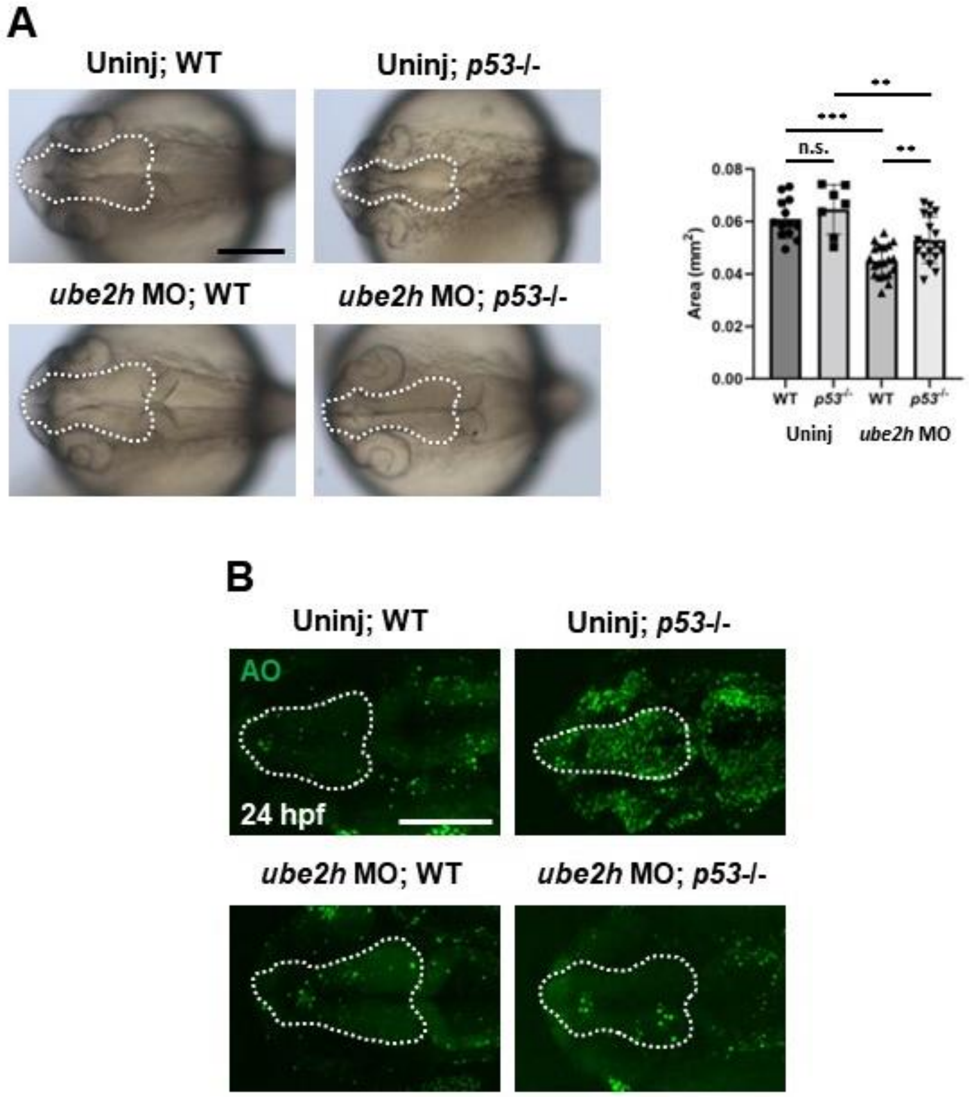

Supplemental Figure S2. Depletion of *p53* restored ectopically induced apoptosis in the *ube2h* morphants. A. Representative dorsal view images of *ube2h* morphants and uninjected controls with either WT or $p53^{-/-}$ background at 24 hpf and quantification of brain size (white dotted area). **B**. Confocal microscopy images of AO-stained brains (white dotted area) of *ube2h* morphants and uninjected controls with either WT or $p53^{-/-}$ background at 24 hpf. The white dotted area indicates the brain organs, including forebrain and midbrain. The graph represents mean ± S.E.M. of individual values. *p*-values were calculated using an unpaired two-tailed Student's t-test. *** $p<0.001$; ** $p<0.01$; n.s., not significantly different. Scale bar: 200μm.

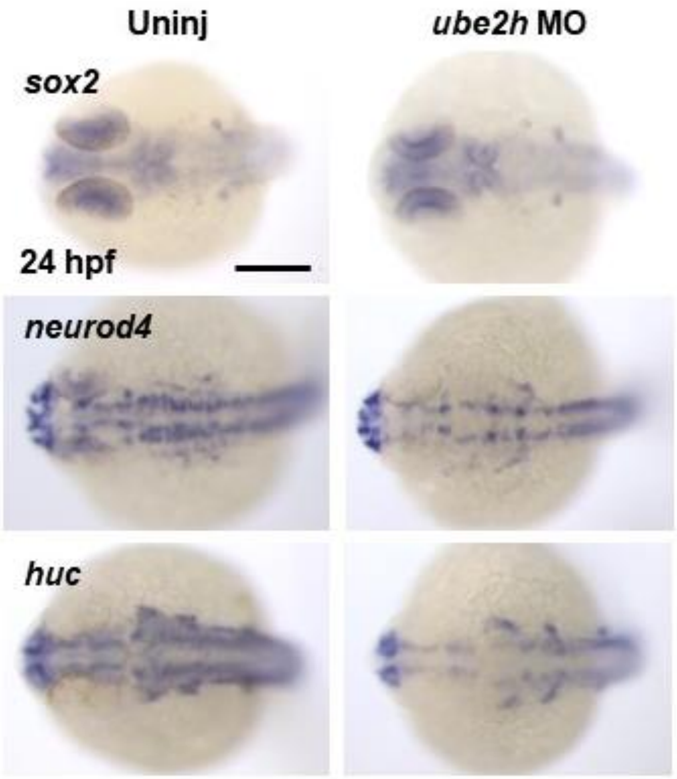

Supplemental Figure S3. Neural marker expression in the *ube2h* morphants. Dorsal view of WISH images using probes for *sox2*, *neurod4,* and *huc* in the brains of *ube2h* morphants and uninjected controls at 24 hpf. Scale bar: 200μm.

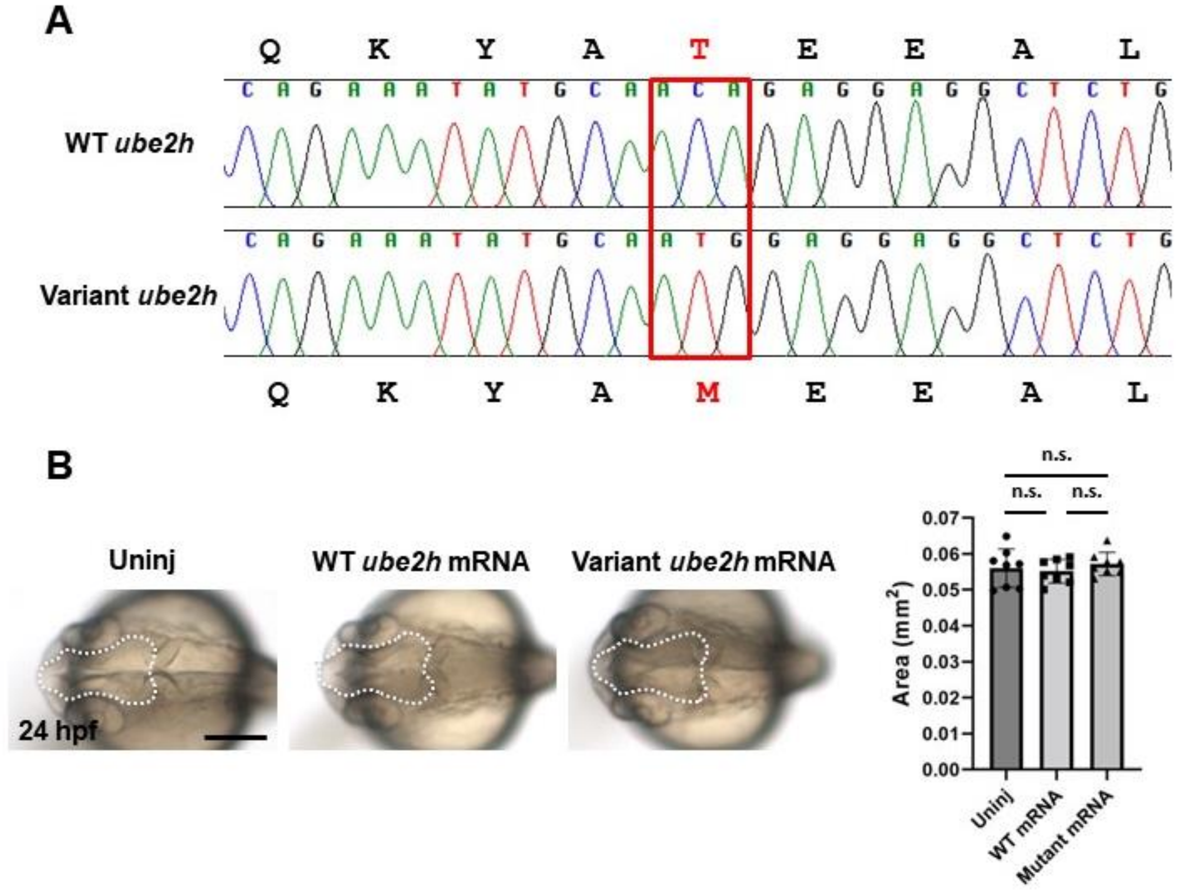

Supplemental Figure S4. Overexpression of zebrafish *ube2h* variant does not alter brain development. A. Sequence of the zebrafish *ube2h* variant [c.449C>T (p.Thr150Met)]. **B**. Dorsal view of the anterior region of zebrafish injected with either normal WT or variant *ube2h* mRNA. The graph shows the quantification of brain sizes in the dotted area of each sample. The graph represents mean ± S.E.M. of individual values. *p*-values were calculated using an unpaired two-tailed Student's t-test. n.s., not significantly different. Scale bar:200μm.

Chromosome	SYMBOL	Variants type	Mode of inheritance	EXON	Genome change	Protein change	IMPACT	SIFT	PolyPhen	ACMG Evidence	1KGP AF	1KGP EAS AF	gnomAD AF	gnomAD EAS AF	Korea1K AF
Chr7	UBE2H	Missense Mutation	*de novo*	7/7	c.449C>T	p.Thr150Met	MODERATE	Deleterious (0.04)	probably_damaging (0.958)	PS2, PM2	0	0	0	0	0

Supplemental Table S1. Information of UBE2H Variants
